# The association between MTHFR polymorphism and cervical cancer

**DOI:** 10.1038/s41598-018-25726-9

**Published:** 2018-05-08

**Authors:** Jiao-Mei Gong, Yong Shen, Wan-Wan Shan, Yan-Xia He

**Affiliations:** 1 Department of Clinical Laboratory, Second Affiliated Hospital of Zhengzhou University, Zhengzhou, Henan China; 2 Department of Clinical Laboratory, Affiliated Cancer Hospital of Zhengzhou University & Henan Cancer Hospital, Zhengzhou, Henan China; 3 Department of Obstetrics and Gynecology, Third Affiliated Hospital of Zhengzhou University, Zhengzhou, Henan China

## Abstract

Cervical cancer is an extremely prevalent disease worldwide. The purpose of this study was to illustrate the relationship between methylenetetrahydrofolate reductase (MTHFR) polymorphisms or methionine synthase reductase (MTRR) polymorphisms and cervical cancer. There were 372 women who performed genetic and folic acid assessments. For the MTHFR C677T, there was no significant difference in the distribution of C allele and T allele in the three groups. However, the mutant C allele of MTHFR A1298C was significantly higher in the cancer group than in the normal group. Similarly, the mutant G allele of MTRR A66G was also higher than the normal group. The serum folic acid levels were gradually decreased with the development of cervical lesions. Serum folate levels in 4–9 ng/ml and ≤4 ng/ml were both significantly associated with cervical cancer risk. However, the MTHFR C677T polymorphism was not associated with the risk of cervical cancer or CIN. In contrast, the MTHFR A1298C polymorphism could increase the risk of both cervical cancer and CIN. In addition, the MTRR A66G polymorphism was only associated with the risk of cervical cancer but not CIN.

## Introduction

Cervical cancer is an extremely prevalent disease worldwide, representing 13% of female cancers. The incidence of cervical cancer is second among the cancers affecting women^1^. The mortality rate is lower than the incidence, with approximately 275,000 deaths in 2008 worldwide^2^. Moreover, there were 86% of new cervical cancer cases occurring in developing countries^3^. However, the mortality rate was greatly increased, resulting from the relatively late cancer stage of discovery^4^. The health and lives of the women were affected by the condition.

Folate acid is known as vitamin B9 which was necessary for the body to make amino acids, RNA and DNA^5^. As human could not make folic acid which must be obtained from the diet. Therefore, folic acid was an essential vitamin. Folic acid deficiency can occur when the body needs increased folic acid, folic acid in the diet is insufficient, or the body excretes more folate acid than usual. Folate acid deficiency may increase the risk of cancer, such as prostate cancers, colorectal cancer, ovarian cancer, lung cancer, pancreas cancer, cervical cancer and breast cancer^6,7^.

Methylenetetrahydrofolate reductase (MTHFR), encoded by the MTHFR gene, is the rate-limiting enzyme in the metabolism of folic acid. MTHFR catalyzes the conversion of 5,10-methylenetetrahydrofolate acid into 5-methyltetrahydrofolate acid which is a major circulating form of folate acid. The single nucleotide polymorphisms (SNPs) in the MTHFR gene: C677T (rs1801133) and A1298C (rs1801131) had functional relevance to alter the enzyme activity^8^. Thus, MTHFR variation may be associated with variable folate acid levels in folate acid metabolic pathway. It was suspected to be related to cancer risk. Methionine synthase reductase (MTRR) was a flavoprotein that could maintain the methionine synthase enzyme in an active state. The SNPs of MTRR gene: A66G (rs1801394) can decrease MTRR affinity to methionine synthase (MTR)^9^. Thus, it may cause carcinogenesis.

For this study, we performed a large cohort study to evaluate the efficacy of folic acid supplementation and polymorphism MTHFR on developing cervical cancer in China. We anticipate that this report could provide epidemiological evidence and guiding significance of cervical cancer for folate acid supplementation.

## Results

### Patient Characteristics

Among the 372 participants in this study, 116 patients with CIN and 146 cervical cancer patients were compared with the 110 normal women, respectively. The mean age was 40.6 ± 8.2 years in the normal group, 41.6 ± 7.8 years in CIN group and 41.4 ± 9.5 years in cancer group.

### Distribution of genotypes and allelic frequencies

**Figure 1:**
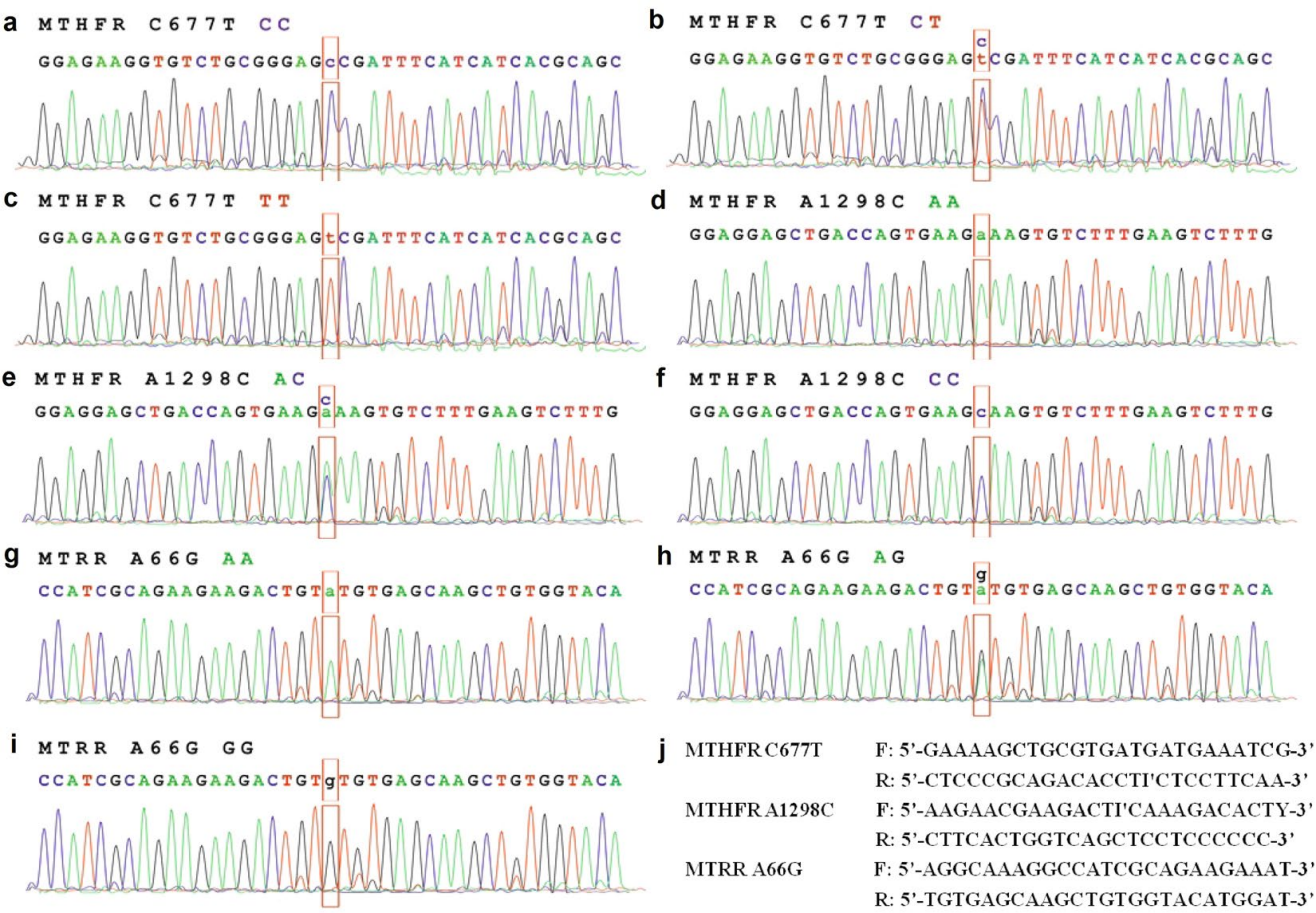
Distribution of MTHFR C677T, A1298C and MTRR A66G genotypes. (**a**) wild-type CC genotype of MTHFR C677T, (**b**) heterozygous CT genotype of MTHFR C677T, (**c**) homozygous mutant TT genotype of MTHFR C677T, (**d**) wild-type AA genotype of MTHFR A1298C, (**e**) heterozygous AC genotype of MTHFR A1298C, (**f**) homozygous mutant CC genotype of MTHFR A1298C, (**g**) wild-type AA genotype of MTRR A66G, (**h**) heterozygous AG genotype of MTRR A66G, (**i**) homozygous mutant GG genotype of MTRR A66G, (**j**) the primers of MTHFR and MTRR.

There are nine genotypes in this study (Fig. 1). Among the nine genotypes, there were three genotypes in MTHFR C677T: CC, CT and TT. In MTHFR C677T, the CC genotype was wild-type (Fig. 1a) and the TT genotype was homozygous mutant (Fig. 1c). The CT genotype was the heterozygous mutant in MTHFR C677T (Fig. 1b). Similarly, the wild-type AA (Fig. 1d), heterozygous mutant AC (Fig. 1e) and homozygous mutant CC (Fig. 1f) were in MTHFR A1298C. Also, there were three genotypes in MTRR A66G: wild-type AA (Fig. 1g), heterozygous mutant AG (Fig. 1h) and homozygous mutant GG (Fig. 1i).

**Figure 2:**
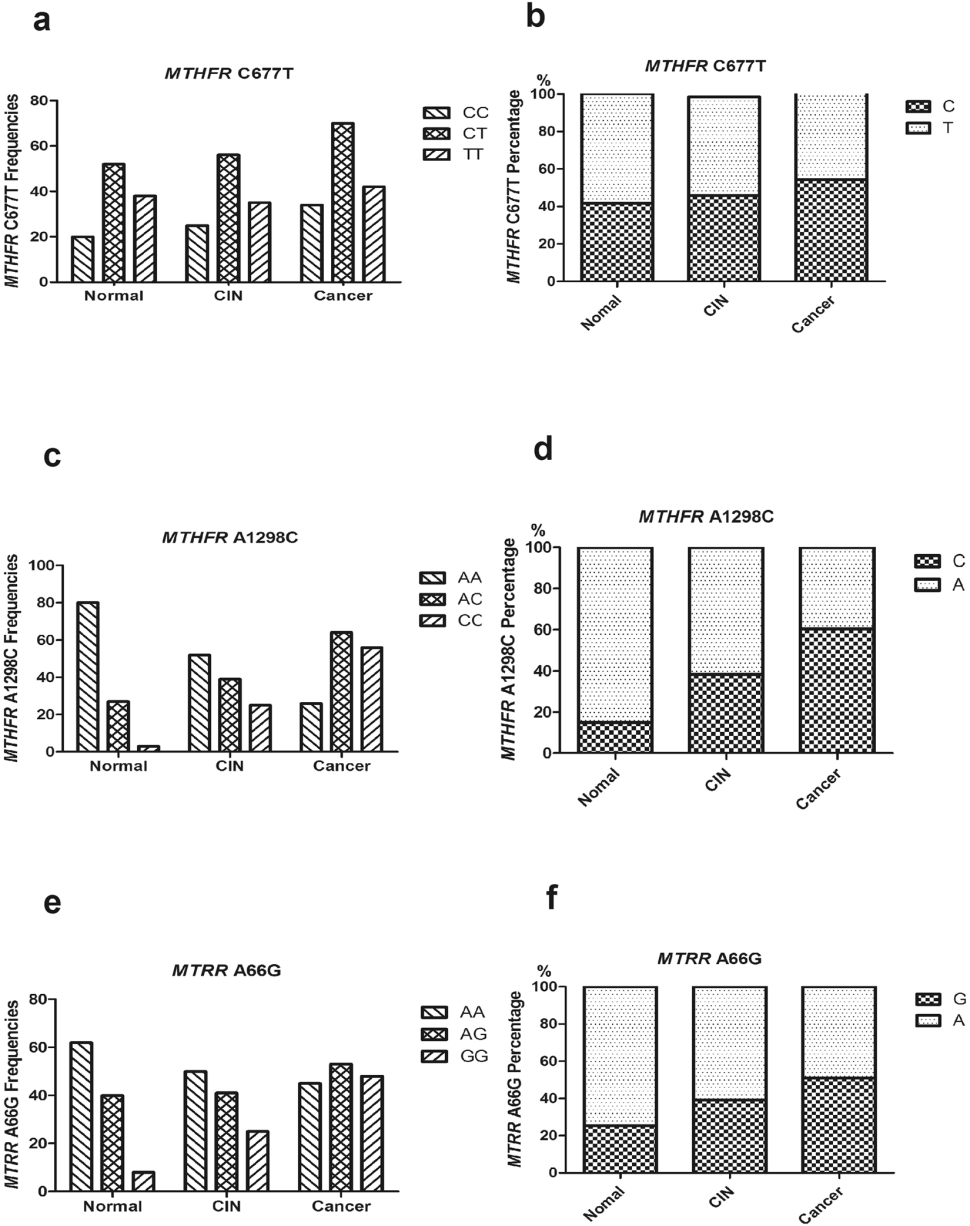
Distribution of MTHFR C677T, A1298C and MTRR A66G allele frequencies. (**a**) the frequencies of CC, CT and TT genotype in MTHFR C677T in normal group, CIN group and cancer group, respectively, (**b**) the percentage of C and T allele in MTHFR C677T in the three groups, respectively, (**c**) the frequencies of AA, AC and CC genotype in MTHFR A1298C in the three groups, respectively, (**d**) the percentage of A and C allele in MTHFR A1298C in the three groups, respectively, (**e**) the frequencies of AA, AG and GG genotype in MTRR A66G in the three groups, respectively, (**f**) the percentage of A and G allele in MTRR A66G in the three groups, respectively.

The distributions of MTHFR C677T, MTHFR A1298C and MTRR A66G genotypes were in Hardy-Weinberg equilibrium. The frequencies of allelic and genotypes in normal group, CIN group and cancer group were presented in Fig. 2. For the MTHFR C677T genotype, there were 20 cases of wild-type CC, 52 cases of heterozygous mutant CT and 38 cases of homozygous mutant TT in normal group, 25 cases of CC, 56 cases of CT and 35 cases of TT in CIN group, and 34 cases of CC, 70 cases of CT and 42 cases of TT in cancer group, respectively (Fig. 2a). Frequency of C allele in the locus of MTHFR C677T was 41.82%, 45.69% and 47.26%, respectively, and T allele frequency was 58.18%, 54.31% and 52.74% in the three groups, respectively (Fig. 2b). Similarly, there were 80 AA genotypes, 27 AC genotypes and 3 CC genotypes in normal group, 52 AA, 39 AC and 25 CC in CIN group, and 26 AA, 64 AC and 56 TT in cancer group for MTHFR A1298C genotypes, respectively (Fig. 2c). Frequency of A allele of MTHFR A1298C was 85.0%, 61.64% and 39.73%, respectively, and C allele frequency was 15.0%, 38.36% and 60.27% in the three groups, respectively (Fig. 2d). For the MTRR A66G genotype, there were 62 AA, 40 AG and 8 GG in normal group, 50 AA, 41 AG and 25 GG in CIN group, and 45 AA, 53 AG and 48 GG in cancer group for MTHFR A1298C genotype, respectively (Fig. 2e). The A allele frequency was 74.55%, 60.78% and 48.92%, respectively, while the G allele frequency was 25.45%, 39.22% and 51.03% in the three groups, respectively (Fig. 2f).

### Association of genetic polymorphisms with cervical cancer risk

**Figure 3:**
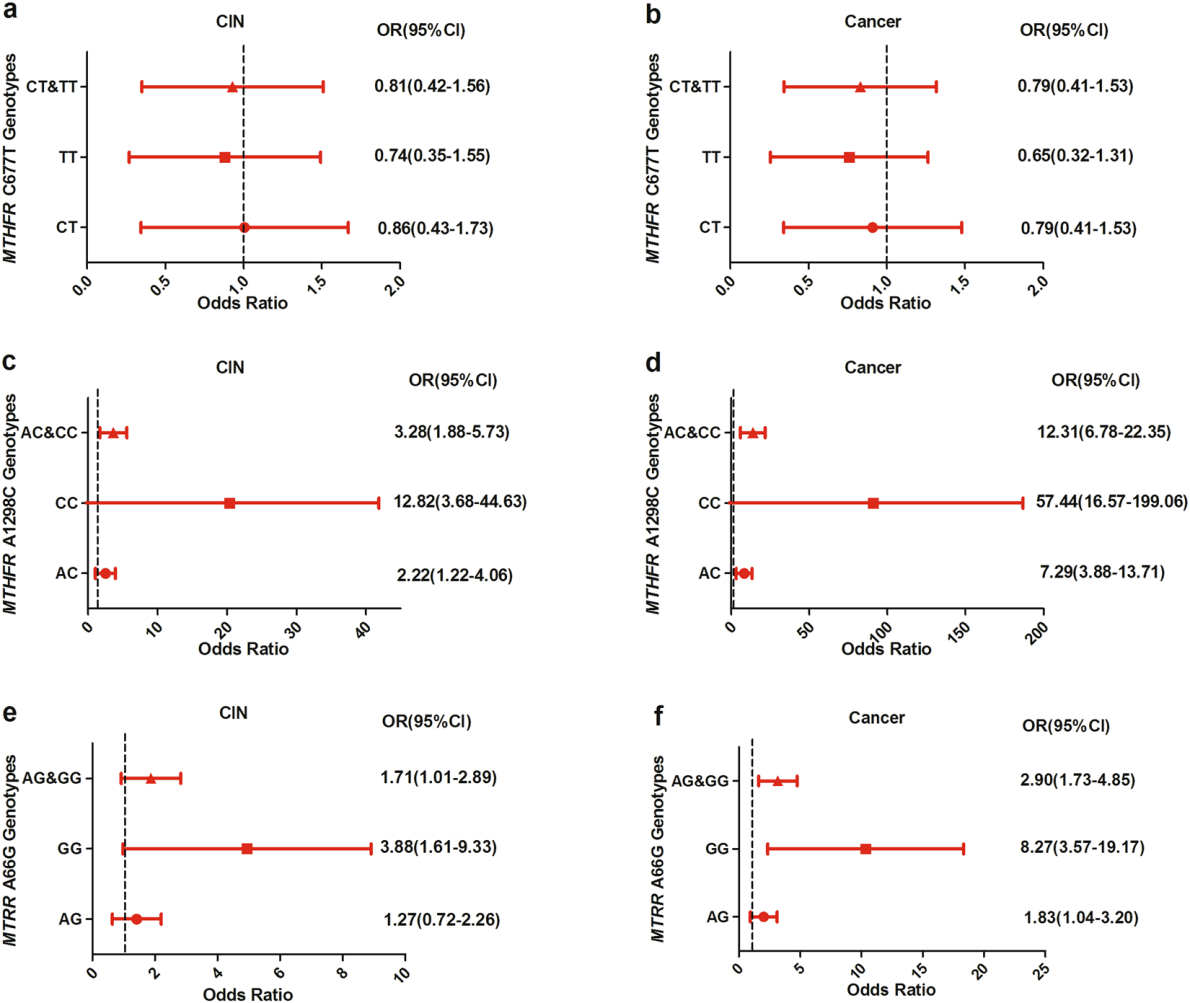
Association of MTHFR and MTRR polymorphisms with risk of cervical cancer. (**a**) MTHFR C677T genotypes associated with CIN, (**b**) MTHFR C677T genotypes associated with cervical cancer, (**c**) MTHFR A1298C genotypes associated with CIN, (**d**) MTHFR A1298C genotypes associated with cervical cancer, (**e**) MTRR A66G genotypes associated with CIN, (**f**) MTRR A66G genotypes associated with cervical cancer.

The association among MTHFR C677T, MTHFR A1298C and MTRR A66G polymorphisms with risk of cervical cancer was presented in Fig. 3. The normal group was used as control. For the MTHFR C677T genotypes, the odds ratio (OR) and 95% confidence interval (CI) of wild-type CC was 1.0. Almost no significant association with risk of CIN were observed among CT genotype (OR:0.86, 95% CI:[1.43–1.73], *P* = 0.86), TT genotype (OR:0.74, 95% CI:[0.35–1.55], *P* = 0.74) and CT&TT genotypes (OR:0.81, 95% CI:[0.42–1.56], *P* = 0.81), respectively (Fig. 3a). Similarly, there is no significant correlation between other MTHFR C677T genotypes (CT, TT or CT&TT) and cervical cancer risk (OR:0.79, 95% CI:[0.41–1.53], *P* = 0.49), TT genotype (OR:0.65, 95% CI:[0.32–1.31], *P* = 0.23) and CT&TT genotypes (OR:0.73, 95% CI:[0.40–1.36], *P* = 0.32), respectively (Fig. 3b). For the MTHFR A1298C genotypes, the wild-type AA was as a contrast. The homozygous mutant CC genotype could increased the cervical cancer risk by 57 times (OR:57.44, 95% CI:[16.57–199.06], *P* = 0.000). While a strong significant association was also observed with cervical cancer risk between AC genotype (OR:7.29, 95% CI:[3.88–13.71], *P* = 0.000) and AC&CC genotypes (OR:12.31, 95% CI:[6.78–22.35], *P* = 0.000), respectively (Fig. 3d). Similarly, there were associations of AC genotype (OR:2.22, 95% CI:[1.22–4.06], *P* = 0.009), CC genotype (OR:12.82, 95% CI:[3.68–44.63], *P* = 0.000) and AC&CC genotypes (OR:3.28, 95% CI:[1.88–5.73], *P* = 0.000) with risk of CIN, respectively (Fig. 3c). For the MTRR A66G, the wild-type AA was as a contrast. A strong significant association was also observed with risk of cervical cancer among GG genotype (OR:8.27, 95% CI:[3.57–19.17], *P* = 0.000), AG genotype (OR:1.83, 95% CI:[1.04–3.20], *P* = 0.04), and AG&GG genotypes (OR:2.90, 95% CI:[1.73–4.85], *P* = 0.000), respectively (Fig. 3f). GG genotype (OR:3.88, 95% CI:[1.61–9.33], *P* = 0.003) and AG&GG genotypes (OR:1.71, 95% CI:[1.01–2.89], *P* = 0.047) were associated with CIN, respectively. However, there was no association between AG genotype (OR:1.27, 95% CI:[0.72–2.26], *P* = 0.41) and CIN (Fig. 3e).

### Efficacy of folic acid with cervical cancer risk

**Figure 4:**
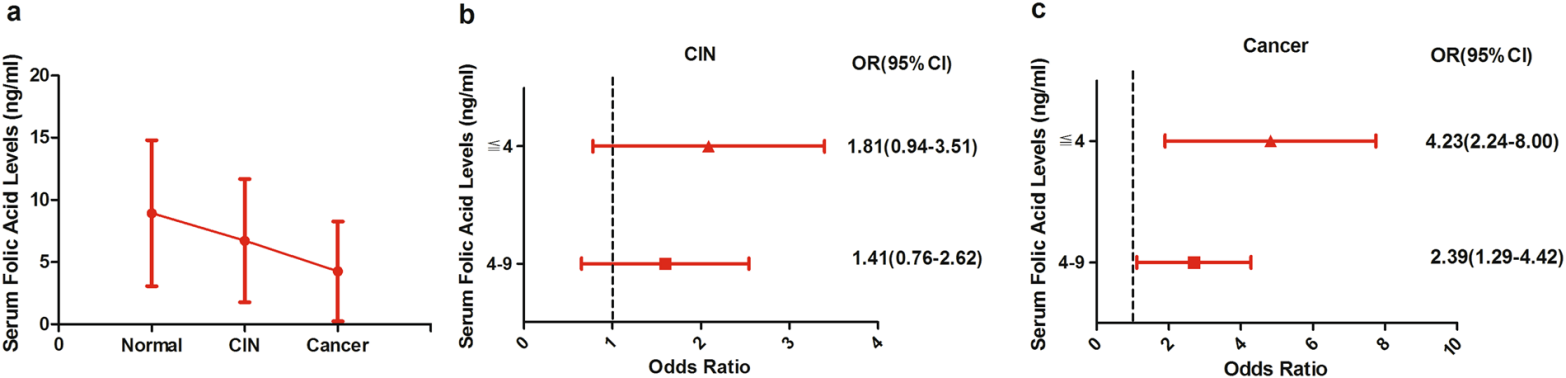
Efficacy of folic acid with risk of cervical cancer. (**a**) the expression levels of serum folic acid in the normal group, CIN group and cancer group, respectively, (**b**) serum folic acid levels associated with CIN, (**c**) serum folic acid levels associated with cervical cancer.

The folate acid metabolism ability was further associated with the MTHFR and MTRR genotypes of cervical cancer. The serum folic acid levels in the normal group, CIN group and cervical cancer group were 8.74 ± 5.86 ng/ml, 6.72 ± 4.95 ng/ml and 4.25 ± 4.03 ng/ml, respectively. The serum folic acid levels were gradually decreased with the development of cervical lesions (Fig. 4a). There was a statistically significant difference between normal group and cervical cancer group (*P* = 0.000).

Compared to the normal group, the OR and 95% CI of serum folic acid ≧9 ng/ml level was 1. In CIN group, the serum folic acid 4–9 ng/ml level did not have apparent effects on CIN incidence specific to participants(OR:1.41, 95% CI:[0.76–2.62], *P* = 0.28) (Fig. 4b). However, there was a statistically significant difference for the serum folic acid ≦4 ng/ml level(OR:1.81, 95% CI:[0.94–3.51], *P* = 0.08) in CIN group. In the cervical cancer group, there was a significant association with risk of cervical cancer with both serum folic acid 4–9 ng/ml level and ≦4 ng/ml level (OR:2.36, 95% CI:[1.29–4.42], *P* = 0.005; OR:4.23, 95% CI:[2.24–8.00], *P* = 0.000, respectively (Fig. 4c).

## Discussion

Cervical cancer is the second most common cancer for women in the worldwide^10^. The incidence of cervical cancer was higher in underdeveloped countries than developed countries because of the result of cervical cancer screening program^1^. Although the cervical cancer carcinogenesis mechanism was not entirely clear. there seem to be some factors, such as genetic factors and HPV infection, that may cause cervical cancer^11,12^. MTHFR and MTRR were key enzymes in folate acid metabolisms. MTHFR gene defects would lead to a number of basic biochemical processes of the body disorders, including cell cycle regulation, DNA replication, DNA methylation modification, which may lead to neural tube defects, cancer, cardiovascular and cerebrovascular diseases^13^.

For the MTHFR C677T polymorphism, some previous studies have been reported the conflicting results associated with the risk of tumor development^14,15^. It can increase the risk of esophageal squamous cell carcinoma (ESCC), ovarian cancer, gastric cancer, colorectal cancer with low folate acid levels and breast cancer^16,17^. However, the risk of colorectal cancer in individuals with homozygous mutant MTHFR C677T TT genotype was observed to be reduced in individuals with sufficient folic acid status^18^. In addition, the MTHFR C677T wild-type CC genotype seems to play a protective role in the development of ESCC in China^19^, whereas the MTHFR C677T CT genotype was associated with a reduced risk of prostate cancer in USA^20^. In this study, the frequency of C allele in the locus of MTHFR C677T was 41.82%, 45.69% and 47.26% in the three groups, respectively, and T allele frequency was 58.18%, 54.31% and 52.74%, respectively. There was no significant difference for the distribution of C allele and T allele in the three groups. Similarly, there was no significant association with a risk of cervical cancer among CT genotype (OR:0.79, *P* = 0.49), TT genotype (OR:0.65, *P* = 0.23) and CT&TT genotypes (OR:0.73, *P* = 0.32), respectively. Some case-control studies had evaluated the association between MTHFR C677T polymorphism and cervical cancer, but the evidence for these studies remained weak^21,22^. In a study, 64 patients with cervical cancer and 31 controls were enrolled^21^. The women with T allele had an increase of 9-fold OR in cervical dysplasia. Another study showed that a variant T allele had doubled in the risk of cervical dysplasia in 150 patients with CIN and 179 controls, and the risk of TT genotype was tripled^22^. The authors also reported that folic acid intake was inversely proportional to the risk of cervical dysplasia. However, some studies had shown that MTHFR C677T polymorphism was not associated with CIN or cervical cancer^23–25^. A study with 21 cervical cancer patients did not find an association with either CIN or cervical cancer^25^. Another study showed a reduced risk of cervical cancer in patients with CT genotype and TT genotype^24^. The results of the two studies were similar to our study. As mentioned above, studies of the association of MTHFR C677T polymorphisms with cervical cancer had produced conflicting results which may be due to the low sample sizes, individual genes in complex diseases, environmental factors and random effects.

Considering the association of the MTHFR C677T polymorphism with cervical cancer in different studies, another common polymorphism of the MTHFR gene was known as MTHFR A1298C. MTHFR A1298C polymorphism was associated with decreased levels of MTHFR enzymes, and it may be associated with CIN and cervical cancer^26^. In our study, frequency of A allele of MTHFR A1298C was 85.0%, 61.64% and 39.73% in the three groups, respectively, and C allele frequency was 15.0%, 38.36% and 60.27%, respectively. There was a significant difference between. The homozygous mutant CC genotype increased the risk of cervical cancer by about 57 times (OR:57.44, *P* = 0.000). While a strong significant association was also observed with risk of cervical cancer between AC genotype (OR:7.29, *P* = 0.000) and AC&CC genotypes (OR:12.31, *P* = 0.000), respectively. Similar to this study, there was a significant association with cervical cancer for MTHFR A1298C in Indian^27^. Compared with the MTHFR A1298C AA genotype, there was a four-fold increase in the risk of cervical cancer for MTHFR A1298C CC genotype in that study. Another study also reported the risk between the MTHFR A1298C CC genotype and cervical cancer^28^. However, there were some studies that did not find the significant association exists between MTHFR A1298C polymorphism and cervical cancer^29^. The conclusion was different from our study. One of the studies showed that MTHFR A1298C polymorphism was not associated with cervical cancer but was associated with CIN^29^. This lack of association may be due to the limited amount of samples reflected, which required further study of larger sample sizes to identify this possible association.

Base on our study, we found a strong significant association with risk of cervical cancer for MTRR A66G GG genotype (OR:8.27, *P* = 0.000) and AG genotype (OR:1.83, *P* = 0.04). Then, GG genotype (OR:3.88) was associated with CIN. However, there was no association with CIN for MTRR A66G AG genotype (OR:1.27, *P* = 0.41). Similar to our results, a study reported that the MTRR A66G variant allele was associated with an increased risk of lung cancer^9^. Another study also reported MTRR A66G G allele was significantly higher in cervical cancer patients^30^. However, other studies have shown that the MTRR A66G mutation allele was not associated with cancer risk^31^.

The folate acid metabolism ability was further associated with the MTHFR and MTRR genotypes of cervical cancer^32^. Study which was on food and folic acid supplements showed that cancer risk was associated with adequate folic acid intake^32,33^. In our study, the expression levels of serum folic acid were gradually decreased over the cervical cancer group, and there was a significant association with risk of cervical cancer between both serum folic acid 4–9 ng/ml level and ≦4 ng/ml level. Insufficient long-term intake of folic acid (below the recommended level of 400 μg/day) may increase the risk of prostate cancer, colorectal cancer, lung cancer, brain cancer, cervical cancer, breast cancer, pancreatic cancer, and ovarian cancer^34^. Other studies have shown that excessive dietary supplementation of folic acid may increase the risk of particularly prostate cancer^35,36^. However, another study showed that taking folic acid supplements was not associated with cancer risk^37^.

In conclusion, according to our study, the MTHFR C677T polymorphism was not associated with the risk of cervical cancer or CIN. However, the MTHFR A1298C polymorphism could increase the risk of cervical cancer and CIN. Also, the MTRR A66G polymorphism was associated with the risk of cervical cancer but not CIN. The low levels of folic acid were also associated with cervical cancer. In addition, larger prospective studies were needed to determine the association between this polymorphism and cervical cancer.

## Materials and Methods

### Study subject recruitment and sample collection

A total of 372 women were enrolled in this study from January 2013 to July 2017 at Second Affiliated Hospital of Zhengzhou University. According to histological diagnosis, participants were divided into three groups: normal group (n = 110), cervical intraepithelial neoplasia (CIN) group (n = 116), and cervical cancer group (n = 146). The inclusion criteria for participants were as follows: age range 28–75 years, non-smoking, no history of other cancers, no immune system disorders and no previous treatment with folic acid.

The study protocols were all approved by the Ethics Committee of the Biosciences Institution of Zhengzhou University. All the participants signed an individual informed consent after receiving a detailed explanation of the research. We confirmed that all methods were performed in accordance with the relevant guidelines and regulations. All identifying information of patient had been removed from all text/figures/tables/images.

### DNA extraction and genotyping of MTHFR and MTRR polymorphisms

The sample DNA was extracted from oral mucosa epithelial cells by column extraction kit (QIAGEN Inc., Valencia, Calif., USA). Every sample DNA was identified the SNPs typing of MTHFR C677T, MTHFR A1298C and MTRR A66G by using TaqMan-MGB SNPs Genotyping Assay (Applied Bio-systems Inc., Foster City, Calif., USA). Fluorescence quantitative PCR was performed for TaqMan-MGB. The primers were shown in Fig. 1j. The PCR amplification conditions were as follows: 95 °C denaturation for 10 min followed by 20 cycles of amplification (92 °C for 15 sec, 60 °C for 60 sec) and 35 cycles of amplification (89 °C for 15 sec, 60 °C for 90 sec). All assays were replicated twice and the genotypic allocation was determined by an automatic allele calling quality value of 0.95.

### Folic acid analysis

Folate acid in serum levels was detected using an electrochemiluminescence immunoassay on an automated Roche Cobas E601 analyzer. The detection limit was determined by analyzing 10 replicates of the zero calibrators and 4 replicate of the lowest nonzero calibrator. The minimum limit of detection of folic acid in serum was 0.64 ng/ml. If the concentration of folic acid was more than 20 ng/ml, the sample was required to be manually diluted and retested. The reference ranges for folic acid was 4.2–19.8 ng/ml.

### Statistical analysis

All statistical analyses were performed using SPSS 17.0 statistical package for windows and GraphPad Prism 5. MTHFR/MTRR genotype frequencies were presented with fisher’s exact test and Chi-square test. Logistic regression analysis was used in estimating the odds ratio (OR), 95% confidence intervals (CI), and the association between MTHFR/MTRR polymorphisms and risk of cervical cancer. All tests were two-sided and interpreted as being significant at P ≤ 0.05.
